# Transurethral resection of the prostate in Northern Nigeria, problems and prospects

**DOI:** 10.1186/1471-2490-8-18

**Published:** 2008-12-06

**Authors:** SU Alhasan, SA Aji, AZ Mohammed, S Malami

**Affiliations:** 1 Department of Surgery, Bayero University and Aminu Kano Teaching Hospital, PMB 3452, Kano, Nigeria; 2 Department of Pathology, Bayero University and Aminu Kano Teaching Hospital, PMB 3452, Kano, Nigeria

## Abstract

**Background:**

Benign prostatic hyperplasia (BPH) is the commonest disease of the urinary tract afflicting the ageing male and is the commonest neoplastic disease in men aged 50 years and above.

Transurethral prostatectomy (TURP) is the ultimate treatment of choice for benign prostatic hyperplasia (BPH) due mainly to the preference of minimally invasive surgery, long term relief of symptoms and cost effectiveness. It is however not available to the majority of Nigerians in need of prostatic surgery in Public Health Institutions.

**Methods:**

The records of patients who underwent prostatectomy in Aminu Kano Teaching Hospital, over the period June 2001 to July 2007 were examined. The bio data of patients and laboratory investigations performed were retrieved.

**Results:**

Five Hundred and forty two patients were operated upon, out of which 40 were excluded due to open prostatectomy (22 patients), bladder neck stenosis (16 patients) or bladder tumour around the trigon (2 patients). The age range of the patients was 47–110 years with a mean of 67.2 years. 289 patients (80.1%) had urethral catheter in situ at presentation and 11 (3%) patients had suprapubic cystostomy of which only 3 (0.85%) had combined urethral stricture and BPH.

Only 131 (26%) had their PSA measured which ranged from 2–100 ng/ml out of which 39(29.8% n = 131) patients had more than 4 ng/ml and cancer of the prostate and 1(0.8%, n = 131) patient had a PSA level of 4 ng/ml and malignant prostate.

Hospital stay was 1–32 days (mean 7.9) and the mean follow up period was 5.6 months (range 0–60) and there were 17.5% complications comprising of urinary tract infection (UTI) 7.2%, Orchitis 2.2%, urinary incontinence 0.6%, atonic bladder 1%, erectile dysfunction 0.6%, cerebrovascular accident 0.4%, myocardial infarction 0.4%, deep vein thrombosis 0.4% and disseminated intravascular coagulopathy (DIC) 0.6% and 1.2% mortality. The cost of treatment inclusive of pre-admission investigations was US$ 615.00 (range US$ 300–1,300)

**Conclusion:**

Despite advances in minimally invasive therapy for LUTH/BPH, TURP is the optimum treatment of choice for the ageing male of sub-Saharan Africa. It is however not available to the majority of patients in this region due to poor health allocation and inadequate facilities and training.

## Background

Benign prostatic hyperplasia (BPH) is the commonest disease of the urinary tract afflicting the ageing male and is the commonest tumour in men over the age of 50 years. Clinically, BPH has been reported to occur in 8% of men at the age of 40 years rising to 50% and 90% by the ages of 60 and 80 years respectively [1]. Although reliable data on the true incidence of prostatic diseases in Northern Nigeria is lacking, patients with BPH form the bulk of urology workload in our centre.

Transurethral resection of the prostate (TURP) is the ultimate treatment of choice of BPH, due mainly to the preference of minimally invasive surgery, long term relief of symptoms and cost effectiveness. It is however not available to the majority of Nigerian patients in need of prostatic surgery in public health institutions, while the high cost in private hospitals is generally prohibitive.

This paper reviews our experience with TURP in Aminu Kano Teaching Hospital, Nigeria and examines the prospects of availing the same in other public hospitals despite limited facilities.

## Methods

The records of patients who underwent prostatectomy in Aminu Kano Teaching Hospital, over the period June 2001 to July 2007 were retrieved and examined. The bio data of patients, records of pre-operative biochemistry, haematological and microbiological investigations performed were also retrieved. Mode of clinical presentation, presence of co-morbidity and its treatment outcome, recorded clinical findings particularly digital rectal examination of the prostate, as well as intra- and post-operative complications and histology of resected prostate chips were also extracted and noted from the patients' case notes and Pathology Department Register respectively.

Informed consents were routinely obtained from all the patients indicating that data recorded in their files could be used for research purposes. The ethical approval was given to all retrospective studies due to its being a form of audit.

Transurethral procedures were carried out by 2 Urological surgeons. Distilled water at a height of 1 metre from the bladder (exerting a pressure head of 98 cmH_2_O) was used as irrigation fluid and one set of size 27 FG Olympus continuous flow working sheath with 5° telescopes were used for resection of all the prostates.

All procedures were abandoned once a capsular perforation was made and 15 (3%) of such cases were identified.

Excluded were patients who had open prostatectomy and transurethral bladder neck incisions. SPSS 12.0 was used for statistical analysis of extracted data.

## Results

A total of 542 patients had prostatectomy out of which 40 were excluded due to open prostatectomy (22 cases), bladder neck stenosis (16 patients) or bladder tumour around the trigon (2 patients). Five hundred and two (502) cases were therefore available for analysis.

The age range of the patients was 47–110 years with a mean of 67.2 ± 9.8 SD (Table 1).

**Table 1:** Age distribution (years)

**Age Range**	**Number**	**Percentage (%)**
45–50	14	(2.8%)
51–55	46	(9.2%)
56–60	86	(17%)
61–65	112	(22.3%)
66–70	116	(23%)
71–75	51	(10.2%)
76–80	46	(9.2%)
81–85	10	(2%)
86–90	11	(2.2%)
91–95	4	(0.8%)
96–100	3	(0.6%)
101–105	3	(0.6%)
106–110	1	(0.2%)

**TOTAL**	**502**	**100%**

Two hundred and eighty nine patients (80.1%) had a urethral catheter in situ at presentation, and 11 (3%) patients had suprapubic catheter (SPC) out of which only 3 (0.85%) had combined urethral stricture and BPH, the others had high riding prostate. Seven patients (1.4%) were identified to have had chronic urinary retention of which 3 (0.6%) never recovered detrusor function and remained with urethral catheter more than a year post-operatively.

Significant co-morbidity was found in 147 (29.3%) patients in the form of cardiovascular disease (137 [27.3%] patients), chronic obstructive airway disease (5 [0.99%] patients) and chronic renal failure (5 [0.99%] patients).

Digital rectal examination revealed benign enlarged prostates in 490(97.6%) patients and 12 (2.4%) patients had suspicious prostate carcinoma, confirmed on biopsy.

**Figure 1:**
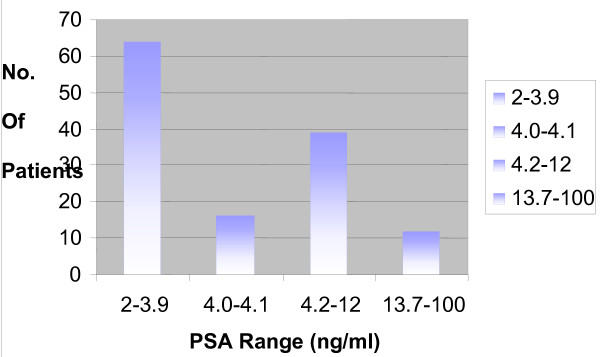
**PSA levels by age groups.** Note that all the confirmed carcinoma of the prostate are in the 13.7—100 range.

Prostate specific antigen (PSA) assay and transrectal ultrasound (TRUS) facilities were not available in AKTH until late 2003 and 2007 respectively. One hundred and thirty one (131 [26%]) patients had PSA assay measured (Figure 1). There was no data available on post void residual volume.

The pre-operative Haemoglobin (Hb) of patients was 5–17 g/dl (mean 12.7 ± 2.2 SD) and 154(30.7%) patients with Hb ≤ 10 were transfused pre-operatively. The post operative transfusion rate for all groups was 0.8%.

Medical treatment in the form of Prazocin 4–8 mg (5 patients, 1%) or alfuzocin 10 mg(8 patients, 1.6%) nocte were given to only 13 patients(2.6%) and of this 3 patients(0.6%) in Prazocin group opted for TURP due to intolerable postural hypotension while remaining 2 patients and those in alfuzocin group(totalling 2%) opted for TURP due to cost consideration long term.

Only 71 patients (14%) had IPSS scored in their files, of which 18 patients (3.6%) had a mean IPPS of 14 ± 3.81 SD and 53 patients (10.6%) had IPSS of 24 ± 7.71 SD. Fourteen patients were not scored for IPSS.

Majority of patients (92%) were operated upon under spinal sub-arachnoid block while 6.7% were done under 'saddle' block, using 3 mls of 0.5% bupivacain and 5 patients (1.3%) had general anaesthesia either as conversion (2 patients) or due to sub-arachnoid blockade contra-indications (3 patients).

The amount of prostate chips resected weighed between 3–146 gm (mean 59.8 gm, ± 27.8 SD) over an operation period of 20–120 minutes (mean 64.1 minutes). There was no documented TUR syndrome.

Urethral catheters were removed in 0–30 days (mean 3.8, ± 4.6 SD) but 10 patients (2%) needed re-catheterization, with successful trial without catheter in 7 patients (1.4%) after 2 weeks. Persistent irritative symptoms were recorded in 2 patients (0.4%) who did not improve on medical therapy at one year follow up. Re-operation was performed in 5 patients 2 years after initial operation due to intractable haematuria (2 patients, 0.4%), recurrent adenoma 3 patients (0.6%). All patients had residual urine measured with a urethral catheter before discharge. Satisfactory outcome was recorded in the immediate post-operative period in 483 patients (96.2%).

There were 6 peri-operative deaths (1.2%) in the age groups 56–60 years (DVT/PE/DIC) and 71–75 years (MI). The 2 myocardial infarctions occurred intra-operatively from hypotensive episodes and 3 patients (0.8%) had chronic renal failure due to obstructive uropathy not resolved by TURP.

Hospital stay was 1–32 days (mean 7.9 ± 5.2 SD) and the follow up period was 0–60 months, (mean 5.6 ± 8.3 SD).

The average cost of treatment inclusive of pre-admission work-up investigations was US$300–1,300, (mean US$617.00 ± 231 SD).

## Discussion

Transurethral prostatectomy is still a novel procedure in most government hospitals in Sub-Saharan Africa due mainly to non-availability of equipments and inadequate number of trained personnel conversant with the procedure. Patients' perception of high post-operative morbidity and mortality associated with open prostatectomy in t he locality makes the majority of them reluctant to present early for appropriate therapy as evidenced by the large number presenting with urethral catheter in situ in our series (83.1%) compared with 24% in North America and 42% in the United Kingdom [2]. The high incidence of urethral catheterisation is known to be associated with 100% bacteriuria and urinary tract infections (UTI) when catheter remains in situ for ≥ 4 days [9]. Detected and treated urinary tract infections resulted in persistent bacteriuria in 9.4% of our patients (Table 2), which is lower than what was reported by Wilson et al (36%) [2,3].

**Table 2:** Urinary microbial isolates in patients with catheter pre-op.

Urine microbial	Pre-operative	Post-operative
Escherichia coli	21 (4.2%)	13 (2.6%)
Klebsiella species	20 (4%)	12 (2.4%)
Proteus species	12 (2.4%)	1 (0.2%)
Staphylococcus aureus	17 (3.4%)	10 (2%)

The highest incidence of LUTS/BPH was found in the age group 56–70 years accounting for a total of 62% of the patients operated in this study. This correlates similar figure reported by Garraway et al of 66% [3,4].

Co-morbidity is both a strong predictor of length of stay and excess risk of death peri-operatively. Co-morbidities of 29.3% in this study compares favourably to 25% for open prostatectomy in Sicilian-Calabrian region of Italy which may suggest similar standards of healthcare in the 2 regions [5].

Water was used as irrigation fluid during resection due to non-availability of glycine and other irrigation fluids which were expensive to import. Distilled sterile water is cheap, available in abundance and safe as irrigation fluid in transurethral resections and though not proven in this study, the intravesical pressure was likely to be less than 40 cmH_2_O which is the critical pressure above which fluid is significantly absorbed [6-8]. There was no accurate record on the number of capsular perforations but they must have occurred in excess of the 15 recorded cases in this series but without serious consequences as regards TUR syndrome, haemolysis or significant haemorrhage.

The complication rate of 17.5% and mortality of 1.2% in this study (Table 3) are rather high, but men presenting with BPH in retention are known to have an excess risk of death and increased risk of peri-operative complications. The mortality rate of 1.2% is higher than that of a larger series reported by Ansari et al from Victorian Hospitals of 0.8% and the widely accepted mortality rate of 1% associated with TURP [9]. This may be due to less optimum back up facilities in our unit and the initial inexperience in the choice of anaesthetic techniques. 'Saddle' sub-arachnoids blockade was introduced in 34 patients (6.7%) in an attempt to reduce the frequently encountered hypotension due to T_10–12 _sympathetic ganglion blockade and none of these suffered similar complications.

**Table 3:** Significant peri-operative complications

Complications	No. (%)	Outcome
Post-operative UTI	36 (7.2%)	Treated
Orchitis	11 (2.2%)	Resolved
Urine incontinence	3 (0.6%)	2 resolved
Atonic bladder	5 (1%)	3 resolved
Erectile Dysfunction	3 (0.6%)	Permanent
Cerebrovascular Accident	2 (0.4%)	2 Recovered
Myocardial Infarction	2 (0.4%)	2 died
DVT/PE	2 (0.4%)	2 died
DIC	3 (0.6%)	2 died
		
**TOTAL**	**57 (13.4%)**	**9 (1.8%) permanent**

Key:

DVT: Deep Vein Thrombosis

PE: Pulmonary Embolism

DIC: Disseminated Intravascular Coagulopathy

Total blood loss was low with a post operative transfusion rate of 0.8% which is better than the rate of 8.2% for open prostatectomy [10-12]. This is an advantage in a society with high incidence of HIV/AIDS and other viral transmissible infections.

TURP is associated with a post-operative hypercoagulable state just as in other pelvic surgeries and a deep vein thrombosis (DVT) of 6.8–10% [13] but most urologists in the locality are apprehensive of using low molecular weight heparin despite this. There were 0.4% patients with DVT in our review ending in fatalities. In spite of this, only physical measures and clotting profile assays were employed to reduce the risk of DVT and pulmonary embolism.

Incidental adenocarcinoma of the prostate of 5.6% (Table 4) is much lower than was found by Dawam (10%) in Zaria [14], due to better assessment by combining digital rectal examination with quantitative prostate specific antigen assay and transrectal ultrasound.

**Table 4:** Prostate histology

Histology	No. of Cases	Percentages (%)
BPH	458	91.2
Incidental adenocarcinoma	28	5.6
Channel TURP (adenocarcinoma)	12	2.4
Incidental Bladder neck SCC	4	0.8
		

**TOTAL**	**502**	**100**

Key: SCC = Squamous cell carcinoma

The predictors of length of stay are: intrinsic patient factors such as co-morbidities, advances in peri-operative care and surgical techniques and extrinsic hospital factors such as resources, capacity and efficient bed management [15]. The length of stay in our study was governed by deficiencies in all of the above factors. Lack of updated equipments and skilled manpower coupled with patients with multiple co-morbidities was the usual scenario. We were able to show positive correlation between co-morbidities and length of stay in this study (Pearson's correlation 0.233, p ≥ 0.01).

Impotence is reported to occur, in the literature in 11% of cases post-TURP, but only 0.6% was picked up in this study due to incomplete information, which is one of the draw backs of retrospective study.

The cost of treatment is rather high for an average Nigerian as the gross domestic product (GDP) per capita of US$800 is low even within Africa, coupled with poor health allocation from the Budget (2.6%) [16].

## Conclusion

The morbidity and mortality of TURP is expectedly higher than what is obtained internationally, but still lower than what was found following open prostatectomy even in European series. The overall cost of TURP was high for an average Nigerian considering the per capita income of US$800:00 but was worthwhile in view of its inherent minimal trauma, short hospital stay and early recovery. Despite advances in minimally invasive therapy for lower urinary tract symptoms/BPH, TURP is the optimum treatment of choice for BPH in the ageing male population of sub-Saharan Africa, but better facilities and skilled medical staff are needed to make it safer and affordable for the majority of patients in public health institutions.

## Competing interests

The authors declare that they have no competing interests.

## Authors' contributions

SUA operated on majority of the patients, conceived of the idea of reviewing the performed prostatectomies over the years and wrote the initial design of the manuscript. SAA operated on some of the patients, collated the data and carried out the statistical analysis as well as critical review of the manuscript AZM reviewed all the histological specimens of the selected patients, participated in the critical review with major corrections of the design of the manuscript. SM reviewed the histology slides with AZM, criticises and made major corrections of the manuscript. All authors read and approved the final manuscript.

## Pre-publication history

The pre-publication history for this paper can be accessed here:


